# Do digital interventions increase adherence to home exercise rehabilitation? A systematic review of randomised controlled trials

**DOI:** 10.1186/s40945-022-00148-z

**Published:** 2022-10-03

**Authors:** Sabine Lang, Colin McLelland, Donnie MacDonald, David F. Hamilton

**Affiliations:** School of Health and Social Care, Edinburgh Napier University, Sighthill Campus, 9 Sighthill Court, Edinburgh, EH11 4BN UK

**Keywords:** Exercise, Adherence, Physical activity, Digital interventions, Systematic review

## Abstract

**Background:**

Home exercise regimes are a well-utilised rehabilitation intervention for many conditions; however, adherence to prescribed programmes remains low. Digital interventions are recommended as an adjunct to face-to-face interventions by the National Health Service in the UK and may offer increased exercise adherence, however the evidence for this is conflicting.

**Method:**

A systematic review was undertaken using MEDLINE and CINAHL databases using the PRISMA guidelines. Randomised controlled trials in any clinical population evaluating the adherence to prescribed home exercise interventions with and without additional digital interventions were included. Publication quality was assessed using the Cochrane Risk of Bias tool.

**Results:**

The search strategy returned a total of 1336 articles, of which 10 randomised controlled trials containing data for 1117 participants were eligible for inclusion. 565 participants were randomised to receive the interventions, and 552 to the control. Seven of the ten trials reported a significant difference in adherence between the control and intervention groups favouring an additional digital intervention. Three trials reported equivalent findings. These three reported longer-term outcomes, suggesting an interaction between adherence and duration of intervention. There was substantial heterogeneity in outcome assessment metrics used across the trials prohibiting formal meta-analysis. This included studies were of low to moderate quality in terms of risk of bias.

**Conclusion:**

The addition of a digital interventions to prescribed home exercise programmes can likely increase exercise adherence in the short term, with longer term effects less certain.

**Supplementary Information:**

The online version contains supplementary material available at 10.1186/s40945-022-00148-z.



## Introduction

The emerging use of digital technology in physiotherapy service delivery has been discussed for years; however, the recent Covid-19 pandemic caused major disruption to face-to-face clinical interactions with most outpatient appointments ceased abruptly in March 2020. There is a new focus on health services operating under a more blended approach, incorporating both face-to-face and digitally augmented appointments and treatments [1].

There is already a well-established use of digital technology to augment clinical management in conditions such as diabetes, chronic obstructive pulmonary disease (COPD), chronic heart failure and cardiovascular disease [2, 3]. Patient centred exercise prescription is a well-recognised therapeutic intervention [4], and home exercise programs are frequently provided to patients as part of clinical rehabilitation or to help self-manage long-term conditions [5]. Completing the recommended exercise prescription and adhering to these exercises brings about long-term benefits, which may include better physical function, reduced pain and being able to reach agreed goals [6]. Long-term adherence can also have a considerable benefit in patients’ quality of life and can have a positive benefit on the economy as it can reduce the burden on healthcare systems, as patients are able to self-manage more effectively [7]. A well-established problem when delivering rehabilitation interventions however is the low adherence rate to home exercise programs [8].

Adherence can be defined as ‘the degree a behaviour corresponds with an agreed-on recommendation’ [9]. It has been reported by Argent et al. [10] that adherence to home exercise programs supplied by physiotherapists could be as low as 50%. Low levels of adherence, specifically mid- to long-term, can limit the effectiveness of rehabilitation and may lead to reoccurrence of injuries or not being able to self-manage long-term conditions, resulting in pain, reduced function and subsequently negative outcomes [11].

Adherence, as a concept, is not well understood by researchers [12]. There are many reasons why a patient may struggle to adhere to exercise programmes, which may include perceived barriers such as not having enough time, work schedules, reduction in self-efficacy, the belief that the treatment is ineffective or seeing early positive results so they then feel they do not need to continue with the treatment [10].

There are many forms of digital technology; from mobile applications to websites to simple phone calls. These technologies can help patients self-manage long term conditions or rehabilitate after an injury by providing education, advice, information, feedback and communication from rehabilitation practitioners, which can in turn enhance a patient’s motivation [13].

The use of digital interventions to improve physical outcomes is well researched area, however, surprisingly, there is little consensus as to whether digital interventions promote exercise adherence in patients undergoing physiotherapy [13]. The aim of this review was therefore to evaluate whether the addition of digital interventions to physiotherapist prescribed home exercise programs for any specified clinical condition improved adherence to these programs.

## Methods

A systematic review of randomised control trials specifically investigating whether the addition of digital interventions can increase adherence to prescribed home exercises was undertaken. The study was reported in line with the Preferred Reporting Items for Systematic Reviews and Meta-Analyses (PRISMA) guidelines [14].

### Search strategy

The search strategy comprised of clusters of terms on exercise, adherence and digital interventions. This was a novel search informed with input from a subject specialist librarian. A detailed search strategy incorporating MESH terms and Boolean operators is provided in Additional file 1: Table S1. MEDLINE and CINAHL were searched from initiation to March 2021. Google scholar and the reference lists of eventually included papers were manually searched for any additional relevant articles.

### Study selection

Randomised controlled trials evaluating a primary outcome of exercise adherence were included. All other study designs were excluded. The interventions accepted were prescribed home exercises for a specified population within a randomised trial framework with an additional digital intervention provided to the intervention group only. A broad definition of a digital intervention was adopted encompassing interventions delivered through communication platforms (such as Zoom) or electronic devices (such as smartwatches), software (such as apps), and through mobile phones (texts or voice calls). The primary outcome of this review was adherence to the prescribed exercises. Adherence was defined as the ‘extent to which a person’s behaviour corresponds with agreed recommendations from a healthcare provider’ [9]. Any measure of adherence was considered (e.g. patient reported) so long as clearly documented as the study primary outcome.

A three-part screening strategy was employed to identify relevant articles. One investigator (SL) carried out the searches and screened by title. Abstracts were reviewed independently by two investigators (SL and DFH) and consensus reached through discussion for full text inclusion. In the event of disagreement, or doubt, manuscripts were included for full text review. Full texts were reviewed by the same two reviewers independently. In the event of unresolvable differing opinions as to final inclusion, an arbitrator (DM) was available for consultation (though was not required).

### Data collection and risk of bias assessment

Data was extracted from the included studies using a bespoke Excel database. Data included year of publication, geographic location where trial was conducted, demographic parameters, condition/clinical presentation, numbers of participants, intervention received, control intervention received, follow-up period and adherence measure utilised. Demographic data was pooled for reporting.

The risk of bias within the randomised control trials was conducted using the Cochrane Risk of Bias (RoB) tool [15]. This tool assess various aspects of study design including randomization, allocation concealment, blinding of participants, personnel and outcome assessors, completeness of data, selective outcome reporting and any other potential biases. The bias was assessed as a judgement of high, medium or low risk. This was independently evaluated by two researchers (SL and DFH).

## Results

### Study selection

**Fig. 1:**
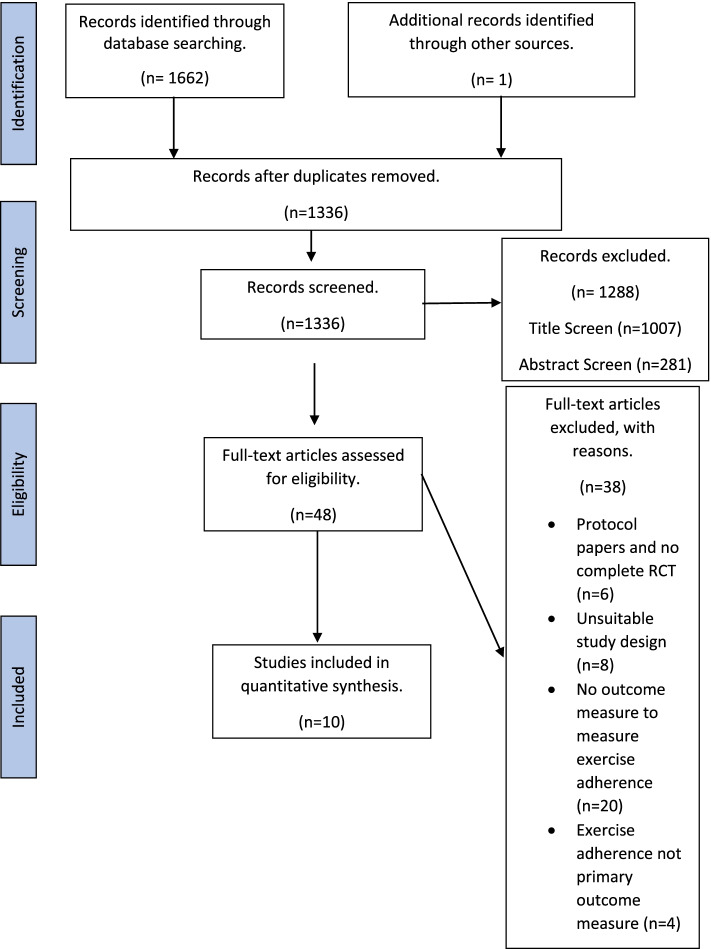
PRISMA diagram showing the search results and article selection process

The search strategy produced a total of 1662 records, 1137 of these were generated from MEDLINE and 525 were generated from CINAHL, a single additional reference from Google Scholar. Removal of duplicates resulted in a total of 1336 articles. After screening, 48 papers were eligible for full text review. Of these 48 papers, 10 met the inclusion criteria (Fig. 1).

### Study characteristics

The main study characteristics and summary of the included study outcomes are presented in Table 1. All studies included within the review were parallel group RCTs. A combined total of 1117 participants were included within the 10 randomised control trials. 565 participants were randomised to receive the interventions, and 552 to the control. The sample sizes for the intervention group ranged from 20 to 152. Of the included studies, 656 (58.7%) of the participants were female, with 461 (41.3%) male. The mean age of participants ranged from 37.5 to 79.5 years.

**Table 1:** Description and summary table of included trials

Author, **year,** country of origin	Clinical Presentation	*n* I/C	Gender m/f	Intervention	Control	Adherence Measure	Follow-up period	Control adherence	Intervention adherence	Difference (*p*-value)
Chung et al. (2020) [16] Hong Kong	Stroke	*56* 27/29	31/25	Home exercise program using a pamphlet, but with additional access to video guided demonstrations of each exercise by scanning QR codes on exercise sheets	Home exercise programme supplied in pamphlet form with pictures and instructions	Self-report, visual analogue scale (0–100)	3-months	55.2%	75.6%	0.021
Bennell et al. (2019) [17] Australia and New Zealand	MSK conditions (including back, shoulder, rotator cuff, knee and hip)	*305* 152/153	127/178	Individualised home exercise program using ‘Physitrack’. Exercises in the system included a description and video of the exercise. Patients could review, record and monitor exercise completion, set up reminders via sms/email and send messages to the physiotherapist	Individualised home exercise program. Included written exercise instructions, exercise logbooks and printed diagrams	Self-report, numerical rating scale (0–11)	3-weeks	6.2	7.3	0.002
Alasfour and Almarwani (2020) [18] Saudi Arabia	Women aged over 50 with knee osteoarthritis	*40* 20/20	0/40	Strengthening exercise program for knee extensors and hip abductors. Additional access to the ‘My Dear Knee’ app, which contained a guide for exercise and send notifications	Strengthening exercise program for knee extensors and hip abductors	Self-report, Exercise logbook	6-weeks	60.19	85.35	0.002
Grau-Pellicer et al. (2019) [19] Spain	Stroke	*34* 21/13	N/A	Multimodal rehabilitation program with supervising adherence to activity through an ‘mHealth’ app, 8-week rehabilitation program including: aerobic, task-oriented, balance and stretching exercises. Two 1-h sessions per week	Conventional remote rehabilitation program	Self-report, ambulatory and sedentary time	3-months	Ambulatory 34.00 min/day Sedentary 9.84 h/day	Ambulatory 90.85 min/day Sedentary 4.40 h/day	0.034 0.012
Van Reijen et al. (2016) [20] The Netherlands	Athletes aged 18–70 who had sustained an ankle sprain within the past 2 months	*220* 110/110	110/110	8-week neuromuscular training programme using an app. App provided the user with instructional videos and verbal instructions. 3 sessions per week, max duration of 30 min each session	8-week neuromuscular training programme using a booklet. The booklet included pictures of the exercises that were to be performed	Self-report, compliance questionnaire	8-weeks	76.7%	73.3%	> 0.05
Chen et al. (2017) [21] Taiwan	Frozen shoulder following corticosteroid injection	60 32/28	23/37	Shoulder exercises prescribed twice per day for a duration of 5-min. Provided with a pamphlet with pictures of the exercises. Additional reminders, encouragement, and educational messages daily for 2-weeks via SMS	Shoulder exercises prescribed twice per day for a duration of 5-min. Provided with a pamphlet with pictures of the exercises	Self-report, two daily questions:	2-weeks	85.2%	96.63%	0.03
Baker et al. (2020) [22] USA	Aged over-50 with knee osteoarthritis	*104* 52/52	19/85	Progressive resistive strength training program delivered in a group exercise class. Twice per week for 6-weeks. Then prescribed BOOST home exercise program. Twice per week for 24-months. Additional BOOST-TLC program which received weekly calls for 6-months, then monthly for the remaining 18-months	Progressive resistive strength training program delivered in a group exercise class. Twice per week for 6-weeks. Then prescribed BOOST home exercise program. Twice per week for 24-months	Self-report, Single item exercise adherence assessment	24-months	4.01	3.63	0.57
Lambert et al. (2017) [23] Australia	Upper or lower limb msk injury or condition	*80* 40/40	28/52	4-week home exercise program. Completed once per day, 3–7 times per week. Participants received exercises via an app. Additional weekly motivational SMS and phone calls	4-week home exercise program. Completed once per day, 3–7 times per week. Participants received exercises on a paper handout	Self-report, numerical rating scale (0–10)	4-weeks	6.5	7.8	0.01
Bennell et al. (2020) [24] Australia	Aged over 50, knee pain on most days of the past month or knee pain for over 3 months	*110* 56/54	36/74	Undertake allocated TARGET prescribed home exercise program unsupervised for 24-weeks. Exercise 3-times per week. Received an automated SMS intervention. Participants received up to 5-messages weekly, frequency reduced over the 24 weeks	Undertake allocated TARGET prescribed home exercise program unsupervised for 24-weeks. Exercise 3-times per week. Received paper-based instructions and an optional logbook to record their sessions	Self-report, Exercise Adherence Rating Scale (Section B)	24-weeks	13.3	16.5	0.01
Svingen et al. (2021) [25] Sweden	Adults post-surgical repair of the flexor digitorum profundus tendon	*101* 52/49	66/35	Standard hand rehabilitation, which included home exercises, information about the injury, restrictions regarding use of hands. Home exercises included active and passive flexion / extension of the interphalangeal joints. Total of 12-weeks. Additional access to an app called ‘BOJSENSKADA’. Including videos of the exercises, notifications, and an exercise diary	Standard hand rehabilitation, which included home exercises, information about the injury, restrictions regarding use of hands. Home exercises included active and passive flexion / extension of the interphalangeal joints. Total of 12-weeks	Self-report, questionnaire at 2- and 6-weeks post-surgery	6-weeks	12.8	11.8	0.123

Three trials were conducted in Australia [17, 23, 24], three in Europe [19, 20, 25], two in Asia [16, 21], one in the Middle East [18] and one in North America [22]. These reflected diverse clinical populations with 10 trials exploring interventions in various musculoskeletal complaints including knee osteoarthritis [18, 22, 24], frozen shoulder [21], ankle sprain [20], flexor digitorium profundus repair [25] and generic musculoskeletal (MSK) conditions [17, 23] and two trials in stroke populations [16, 19]. In all cases, physiotherapists prescribed the interventions as part of a specified rehabilitation program. The digital interventions varied in content, but were primarily communication based, via web interphases, text messages, phone call and phone applications (Table 1). All ten trials provided the control group with standard-of-care physiotherapy treatment for their presenting condition, including a home exercise programme. Data collection timelines varied within the included studies, ranging from 2-weeks to 24-months, with a median follow up time of 17.9 weeks.

### Exercise adherence outcome measures

Exercise adherence was evaluated by self-reported measures in all ten trials. Questionnaires were used by Van Reijen et al., Chen et al., Baker et al., and Svingen et al. [20–22, 25]. Chung et al. [16] used a visual analogue scale (0–100), whereas Bennell et al. and Lambert et al. [17, 23] used an 11-point (0–10) numerical rating scale. Bennell et al. [24] used the exercise adherence rating scale (EARS). Grau-Pellicer et al. [19] measured adherence by participant self-report of their ambulation and sedentary time per day. Alasfour et al. [18] utilised an exercise logbook based on dates of completed exercise sessions. It was not possible to pool outcomes data or to perform meta-analysis due to substantial heterogeneity in assessment tools and outcomes.

### Effects of digital intervention on exercise adherence

Overall, seven of the ten (70%) RCTs recorded a statistically significant positive effect of digital interventions in increasing adherence, with three (30%) demonstrating no between group differences (Table 1).

Four of four (100%) trials that reported short-term follow-up (< 6 weeks) reported a positive effect of a digital intervention. Chen et al. [21] found that shoulder exercise compliance was enhanced (*p* = 0.03) over 2-weeks. Bennell et al. [17] found adherence to a home exercise programme for a range of MSK conditions was enhanced over 3-weeks (*p* = 0.002), and similarly Lambert et al. [23] found an app-based intervention enhanced exercise adherence across a range of MSK conditions over 4-weeks (*p* = 0.01). Alasfour et al. [18] also report greater adherence to a home exercise programme for knee osteoarthritis at 6-weeks (*p* = 0.002).

Two of four (50%) trials found enhanced adherence at 8–12 weeks follow-up. Two MKS based trials found no difference in adherence, Svingen et al. [25] using an app in patients following flexor tendon repair at 8-weeks (*p* = 0.123), and Van Reijen et al. [20] using an app in neuromuscular ankle training at 8-weeks (*p* > 0.05). Conversely, in stroke cohorts, both Chung et al. and Grau-Pellicer et al. [16, 19] found greater adherence to exercise at 12-weeks in groups receiving additional app-based interventions (*p* = 0.021 and *p* = 0.034) respectively.

Two studies reported longer-term timeframes at 24-months, one of which (50%) indicated a benefit of a digital intervention. Bennell et al. [24] report enhanced exercise adherence in people with osteoarthritis of the knee when additional SMS message support was provided (*p* = 0.01), however Baker et al. [22] found no difference in adherence to strength training in elderly patients with knee osteoarthritis using a telephone-based methodology (*p* = 0.57).

### Risk of bias within included studies

**Fig. 2:**
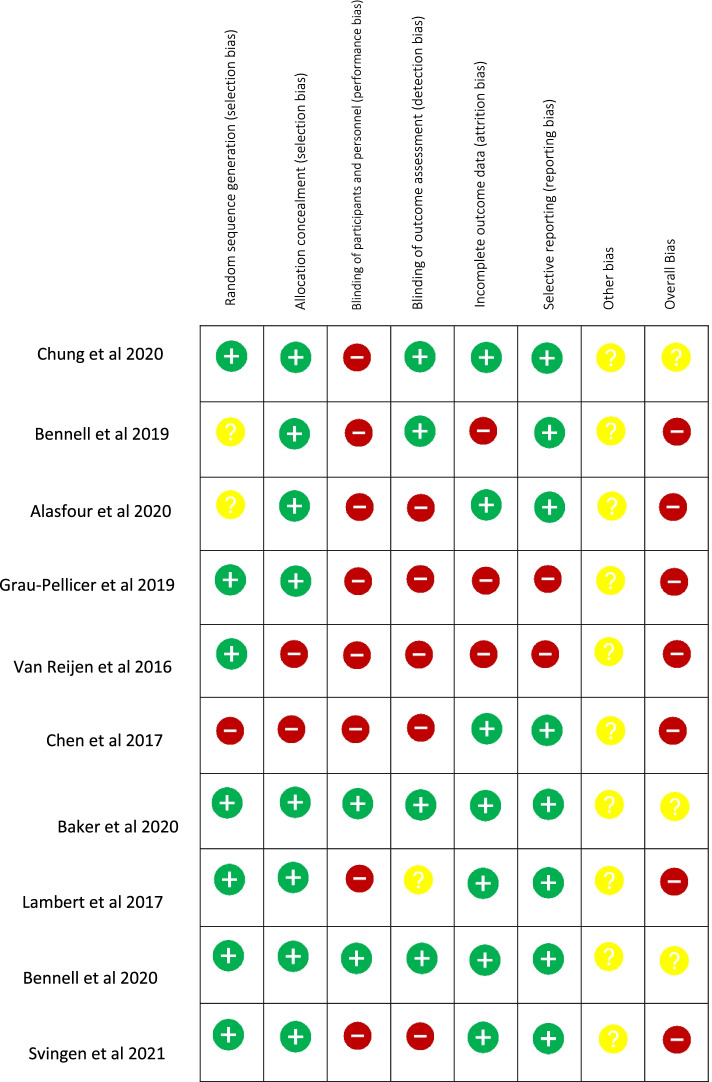
Results of Cochrane risk of bias tool for all included studies

The overall quality of included studies was poor, therefore having a moderate to high risk of bias (Fig. 2). The main sources of bias were related to blinding. Blinding of participants in this type of study is extremely difficult, however blinding of outcome assessors was also poor and the measurement of exercise adherence using self-report tools a concern. Eight of the included studies were at high risk of bias due to not blinding participants and personnel. There were six different self-reported measures used, all with a lack of established reliability and validity estimates. All included studies experienced loss of participants to follow up. Median loss to follow-up was 10.1% with a range of 3.75% to 18.8%. No trial reported a problematic loss to follow-up (accepted at > 20% [26]).

## Discussion

This systematic review of 10 randomised control trials including 1117 participants suggests that digital interventions may help increase exercise adherence to prescribed home exercise plans, at least in the short term, however the data is of low to moderate quality.

There is a growing drive to include digital interventions within physiotherapy practice and treatment [27, 28]. Digital communication interventions have many positives, such as being comparatively simple and cost-effective to use, depending on the technology literacy and physical ability of the target user groups. With an ever-increasing pressure on physiotherapy services, the addition of well-researched digital interventions combined with face-to-face treatments could facilitate efficient clinical management and increase long-term self-management of conditions [28–31].

Within this review, seven of ten included trials suggest increased adherence with digital adjuncts though there may be an association with diminishing returns with prolonged timeframes of use, as the trials that reported the largest benefits of digital interventions tended to have shorter follow-up times. There are many reasons why adherence could reduce over time, though primarily it is accepted that motivation to exercise decreases with time [32]. By prescribing exercise, health professionals are aiming to change a patient’s behaviour. The COM-B Model, developed by Michie et al. [33] for example, states that capability, opportunity and motivation need to be present for a desired behaviour change. Interventions should consider all three components of the model, and incorporate the aspects required depending on the specific barriers present. It is through such feedback and monitoring that physiotherapists and other rehabilitation professionals can help to challenge some of the barriers to behaviour change, for example low self-efficacy or motivation [34]. Interestingly, only one study included in this review [24] reported the behaviour change theory the trial was based on. It is tempting to assume that the inclusion of feedback and monitoring via digital interventions can help to improve exercise adherence, however convincing data for this is lacking. The intervention also needs to be ‘user friendly’ to facilitate regular usage. Many studies reported participants having issues with the technology employed, which may also be a contributing factor to adherence rates in the longer term as patience to engage wears thin [35]. Hypothetically, the mode of communication (or type of digital technology) may influence the client-therapist interaction and thus intervention success; however, the data we obtained in this review is insufficient to ascertain this. The seven articles that reported statistically significant benefits of digital tools involved communication via SMS or email, videos and apps, while the three that reported no difference utilised phone calls, videos and apps. Further comparative trials are required to elicit any effect of specific communication technologies.

Loss to follow-up was not a major concern in the included studies with all ten trials reporting values less than 20%. Despite this, the range of patient attrition was substantial at 3.75% to 18.8%. Perhaps as expected, the studies with the longer follow-up periods tended to report the larger participant loss to follow-up rates [19, 22, 25]. Baker et al. [22] which included the longest follow up time of 24-months, recorded 14.4%, though interestingly the greatest loss to follow-up (18.8%) was reported by Svingen et al. [25] at only 6-weeks. The setting or context of the study may confound the attrition rate, and we observe that successful rehabilitative interventions tend to be discontinued, as they are no longer required. It is notable perhaps that the beneficial effects of digital interventions seen in the (short term, acute setting) Svingen et al. study related to a specific post-operative rehabilitation intervention following finger surgery, whereas the equivocal results of the Baker trial related to much longer-term management of knee osteoarthritis. Importantly there was substantial variation in loss to follow-up in both intervention and control groups. Four trials reported greater attrition in the intervention group [17, 20, 23, 25] while six highlighted greater loss in the control group [16, 18, 19, 21, 22, 24]. This seemingly random loss to follow-up suggests that the addition of a digital intervention was not too onerous or annoying to the patient; however, the heterogeneity in trial content, intervention, timeframe and medical condition may be confounding here.

### Strengths and limitations of the study

This review is the first to evaluate high-level clinical trial evidence as to the effect of technology in increasing adherence to prescribed home exercise interventions. Various others have considered the role of technology in adherence to interventions in condition specific settings and accommodated various trial methodologies [36–38]. Our focus on randomised control trials isolates the effect of the digital intervention and reduces bias, however as there are comparatively few RCTs in this area this increased rigor somewhat limited the source data. Despite this, we are able to report data for over 1000 participants. The heterogeneity of the RCTs included may limit our ability to detect confounding interactions on adherence such as the clinical condition being addressed or factors related to the specific exercise interventions.

Further limitations include our inability to make firm conclusions due to the limited methodological quality of the data. It was not possible to perform a meta-analysis due to the variation in both intervention and outcome measures utilised. The use of self-reported measures of exercise adherence imposes a high risk of bias as the patients may wish to delude themselves as to their compliance with rehabilitation to try to please the physiotherapist. There were numerous methods of measuring exercise adherence employed, however none have been well tested for reliability or validity. This is an ongoing issue in the rehabilitation literature. A review conducted by Bollen et al. [39] found that of 61 different measures of exercise adherence, only two had been appropriately tested as to psychometric properties. We are unable to make any comment as to whether increased adherence improves clinical outcomes as this was not the focus of our study.

## Conclusion

This systematic review of randomised controlled trials of digital intervention to increase adherence to prescribed exercise rehabilitation suggests a short-term beneficial effect, and uncertain longer-term effect. However, the quality of evidence to base these conclusions on is low to moderate.

## Supplementary Information

**Additional file 1: Supplemental Table 1.** Database search strategies.

## Data Availability

The datasets used are available from the corresponding author on reasonable request.
