# Supplementary material for: Do digital interventions increase adherence to home exercise rehabilitation? A systematic review of randomised controlled trials
Source: Arch Physiother. 2022 Oct 3;12:24. doi: 10.1186/s40945-022-00148-z (PMC9527092; doi:10.1186/s40945-022-00148-z)
Supplement: Supplementary file 1 — Additional file 1: Supplemental Table 1. Database search strategies. [file 40945_2022_148_MOESM1_ESM.docx]

**SUPPLEMENTAL FILES**

Supplemental table 1: database search strategies

| **Database** |  | **Search Terms** |
| --- | --- | --- |
| MEDLINE | Exercise Terms | 1. exercis* OR physical activit* |
|  | Adherence Terms | 1. adhere* OR Comply OR compliance |
|  | Digital Technology Terms | 1. Digital or e-health or mhealth or mobile or app or smartphone or cellphone or mobile phone or text messag* or iphone or ipod or ipad or android |
|  | Exercise terms AND adherence terms AND digital technology terms | 1 AND 2 AND 3 |
| CINAHL | Exercise Terms | 1. exercis* OR physical activit* |
|  | Adherence Terms | 1. adhere* OR Comply OR compliance |
|  | Digital Technology Terms | 1. Digital or e-health or mhealth or mobile or app or smartphone or cellphone or mobile phone or text messag* or iphone or ipod or ipad or android |
|  | Exercise terms AND adherence terms AND digital technology terms | 1 AND 2 AND 3 |
